# What Is the Pension Fund Doing?

**Published:** 1893-03-18

**Authors:** 


The Hospital

AN INSTITUTIONAL JOURNAL OF

Science, Medicine, Nursing, and Philanthropy.

*For Hospitals, Asylums, Infirmaries, Dispensaries, Medical Practitioners, Students, Nurses, and the Charitable Public.*

VOL. XIII.—No. 338.] [MARCH 18, 1893.

### WHAT IS THE PENSION FUND DOING?

THE time has come when the Royal National Pension Fund for Nurses may begin to be tested by its fruits. Of opposition by abstract argument, and defence by academic logic it has had enough and to spare. We can now come to close quarters with its actual life and work. The sixth annual meeting was held on Thursday, March 9th, and a plain statement cf facts and figures was placed before the assembled members. Never was a statement of plain facts more interesting or important to the parties concerned.

Let us begin with those circumstances in the year's working which most appeal to our feelings, our unsophisticated humanity! The report tells us, with a "touch of nature," which at once makes us "kin" with the nurses, that some of those young ladies had found themselves impecunious during the year; they had come to the end of their resources; their "last friend" had vanished out of sight; and their "last shilling" was mourning the loss of its companions. We are purposely treating this subject of threatened poverty a little lightly, because we do not think a young and healthy woman should despair when she finds herself alone in the world, and poor in both friends and money. Nevertheless, the situation is not pleasant—far from it; and if the pressure be continued too long despair or temptation is apt to come dangerously near. In such circumstances as these the Pension Fund has proved itself the very friend in need to quite a considerable number of distressed and deserving nurses. It has advanced them loans of money—small sums in most cases—but all they asked, and quite sufficient to carry them "across the bar" of immediate necessity out to the open and smiling sea of work and wages. A novel departure this, but one which is extremely sensible, and which shows that the managers of the Fund, though high financiers and stern economists, are not mere brazen money machines, but kind, thoughtful, resourceful men and women, who sympathise with the youth and inexperience of nurses, and are prepared to act the part of judicious and kindly parents to all of them.

But a large number of nurses have been a great deal too seriously reduced in health and resources for help by mere loans. They have had to stand face to face with starvation. Hunger and homelessness have come within sight, and in some cases almost within touch. No fewer than thirty-three during the single year 1892 have had to receive gifts from the Junius S. Morgan Benevolent Fund. But far more than thirty-three have applied for this kindly aid, and it has been a real distress to the dispensers of that fund that their resources have been so unequal to the demand. The Pension Fund, indeed, is proving itself a candle which lets in light upon many a dark and dreary hiding-place of nursing life. The young, cheery, and neatly uniformed creature who is the life of the sick room, who may be seen soberly walking the streets of our large towns, or demurely waiting among the crowds at our railway stations, shows us one side of the picture. But the picture has its reverse. That same young woman, broken down with work and the confinement of the sick room; ill with some of the thousand-and-one illnesses of which her delicate sex is the subject; hiding herself away in some poor garrett lest those who knew her so bright and hopeful a year or two ago should see her in the desperate straits to which she is now reduced; penniless and homeless—what shall she do? The Pension Fund has helped her to the utmost of its ability! Would that its resources were multiplied fourfold, in order that every deserving nurse who falls into sickness and poverty might be restored to health, hope, and the cheerfulness of daily work.

From a purely business point of view the Royal National Pension Fund has prospered exceedingly during the year. Of new proposals there were 512, and of new pension policies 486 were issued. The sick pay proposals numbered 18[illegible], and the policies issued 168, as against 128 in the previous year. There were no fewer than 89 nurses who received sick pay for a longer or shorter period. These, of course, are not to be included among those who received help from the Benevolent Fund. They are the provident nurses who look ahead, and who by prudent forethought qualify themselves to demand as a right what their less prudent or less fortunate sisters have to plead for as a charity. At the close of the year the Pension Fund and its branches could show invested funds amounting to the substantial, not to say magnificent, total of more than one hundred and thirty-four thousand pounds.

The Pension Fund is thoroughly alive. It has an admirable Chairman in Mr. Walter Burns. He is the soul of judgment and business capacity, and as kind and thoughtful as he is financially distinguished. Of the Deputy-Chairman we are not permitted here to speak, though the writer claims the right to express the opinion that his services during the year demand the special recognition of all the nurses for whom this excellent Fund has been founded. The Founder devoted a considerable part of his much-needed autumn holiday to a missionary tour on behalf of the Fund in the Midlands, the North of England, and Scotland. In the course of that tour he visited Northampton, Leeds, Bradford. Huddersfield, Hull, Durham, Newcastle, Carlisle, Edinburgh, Glasgow, and Aberdeen. Now that tour was taken solely in the interests of nurses and hospitals. It could not add anything to the value of the Pension Fund. The Fund has secured its own continued existence and prosperity by the substantial endowments which are invested on its behalf. The object of the founder was therefore simply to extend the benefits of the organisation to as large a number of nurses and hospitals as possible. The nurses ought to see that what has been done has been done for them; and hospital managers ought also to see that whatever is for the benefit of nurses is for the advantage of hospitals. Let justice be done to every man, though the heavens should fall; and let nurses and hospitals give gratitude where gratitude is due.

The Council of the Pension Fund is one of the most loyal of bodies. It is profoundly interesting to see those grave masters of high finance attending the meetings with unfailing regularity, and devoting themselves to the details of business as if, not unknown nurses but themselves and their children were the objects of their careful solicitude. The matrons, too—those rare and competent managers of vast institutions—lend their best energies to the questions at issue, and stand like a watchful army for the safety and care of the nurses. In short, if an outsider may be pardoned for expressing a strong opinion, the nurses of this country have never before had an institution or friends so capable of helping them at almost any and every crisis of their lives as the Royal National Pension Fund.

It is a real pleasure to the writer to be allowed, on the rare occasion of an annual meeting, to point out to nurses the disinterested regard of which they are the subjects throughout the whole year. The chairman and the council, with their lady advisers the selected matrons, meet month by month, consider proposals, claims, necessities, pleas of poverty, and all the numerous hardships of the nurse's lot. They deal with facts and figures, study the money market, and make safe and profitable investment of nurses' money; they provide for both present and future. And all for what? For the mere satisfaction of helping a class of most meritorious and indispensable women, who but for their help would be entirely at the mercy of every wind of adversity and ill-fortune that blows. Undoubtedly the Pension Fund is a noble institution, and is destined to play a much more pervading and advantageous part in the nursing world than any of us have yet given it credit for.

